# Predictive value of neutrophil to lymphocyte ratio in patients with acute ST segment elevation myocardial infarction after percutaneous coronary intervention: a meta-analysis

**DOI:** 10.1186/s12872-018-0812-6

**Published:** 2018-05-02

**Authors:** Sai Zhang, Jun Diao, Chunmei Qi, Jingjing Jin, Li Li, Xingjuan Gao, Lei Gong, Weiheng Wu

**Affiliations:** 1 Department of Cardiology, The Second Affiliated Hospital of Xuzhou Medical University, Xuzhou, Jiangsu China; 2 The Second Affiliated Hospital of Xuzhou Medical University, 32th Meijian Road, 221000 Xuzhou, Jiangsu People’s Republic of China

**Keywords:** Neutrophil to lymphocyte ratio, ST segment elevation myocardial infarction, Percutaneous coronary intervention, Inflammation, Mortality, Prognosis

## Abstract

**Background:**

The neutrophil to lymphocyte ratio (NLR) is an indicator of systemic inflammation and a prognostic marker in patients with acute coronary syndrome (ACS). This study aims to investigate the value of NLR to predict the in-hospital and long-term prognosis in patients with ST segment elevation myocardial infarction (STEMI) after percutaneous coronary intervention (PCI) by meta-analysis.

**Method:**

The studies related to the prognosis of NLR and STEMI patients published in the Pubmed, Embase, and Ovid databases before June 2017 were retrieved. The relevant data were extracted. Review Manager Version 5.3 was used for meta-analysis.

**Results:**

A total of 14 studies of 10,245 patients with STEMI after PCI were included. A significant difference was observed for mortality (*P* < 0.001; relative risk (RR) 3.32; 95% confidence interval (CI) 2.45–4.49), hospital cardiac mortality(*P* < 0.001; RR 3.22; 95% CI 2.25–4.60), all mortality (*P* < 0.001; RR 3.23; 95% CI 2.28–4.57), major adverse cardiovascular events (MACE) (*P* < 0.001; RR 2.00; 95% CI 1.62–2.46), in-stent thrombosis (*P* < 0.001; RR 2.72 95% CI 1.66–4.44), nonfatal myocardial infarction(MI) (*P* < 0.001; RR 1.93; 95%CI 1.43–2.61), angina (*P* = 0.007; RR 1.67; 95%CI 1.15–2.41), advanced heart failure (AHF) (*P* < 0.001; RR 1.81; 95% CI 1.48–2.21), arrhythmia (*P* = 0.002; RR 1.38; 95% CI 1.13–1.69), no reflow (*P* < 0.001; RR 2.28; 95% CI 1.46–3.57), long-term all mortality (*P* < 0.001; RR 3.82; 95% CI 2.94–4.96), cardiac mortality (*P* = 0.004; RR 3.02; 95% CI 1.41–6.45), MACE (*P* < 0.001; RR 2.49; 95% CI 1.47–4.23), and nonfatal MI (*P* = 0.46; RR 1.32; 95% CI 0.63–2.75).

**Conclusions:**

Meta-analysis shows that NLR is a predictor of hospitalization and long-term prognosis in patients with STEMI after PCI, but requires further confirmation by large randomized clinical trials.

**Electronic supplementary material:**

The online version of this article (10.1186/s12872-018-0812-6) contains supplementary material, which is available to authorized users.

## Background

Inflammation plays a key role in the occurrence and development of atherosclerosis [1]. The neutrophil to lymphocyte ratio (NLR) is an indicator of systemic inflammation and a prognostic marker in patients undergoing percutaneous coronary intervention (PCI) [2, 3]. Furthermore, in previous studies, the NLR has been demonstrated to be related to in-hospital cardiovascular mortality and long-term mortality in patients with ST segment elevation myocardial infarction (STEMI) [4, 5]. However, the results of individual studies were different. Our meta-analysis aimed to evaluate the relationship between NLR and in-hospital or long-term prognosis of patients with STEMI after PCI.

## Methods

### Study search strategy

A systematic search was conducted in the English-language databases of Pubmed, Embase, and Ovid (from inception to June 2017) for studies regarding in-hospital or long-term prognosis of patients with NLR and STEMI after PCI. According to the language of the databases, we used the appropriate search strategy. (“Neutrophils to lymphocytes ratio,” or “NLR” or “neutrophils lymphocytes ratio”) and (“ST Segment Elevation Myocardial Infarction,” or “ST Elevated Myocardial Infarction,” or “STEMI”) and (“Percutaneous coronary intervention,” or “PCI”) in the title or abstract terms were used as key words for the English-language databases.

### Inclusion and exclusion criteria

Studies were included if they met the following criteria: (1) the subjects were STEMI receiving primary PCI; (2) the study types were prospective cohort study or retrospective cohort study; and (3) risk estimates of association between NLR levels and cardiovascular related events occurring during hospital or follow-up were studied experimentally. Tests that did not meet all of the above criteria were excluded.

### Data extraction

Study searches and the data extraction of the included studies were conducted independently by two researchers. The quality of each study was evaluated independently using the Newcastle-Ottawa Scale [6]. This scale uses a star system to evaluate nonrandomized studies regarding 3 criteria: patient selection, comparability of study groups, and outcome assessment. Studies achieving a rating of 6 stars or higher were considered to be of the highest quality. The basic information include domains of authors, year of publication, age of target participant, comparison of NLR, number of events, sample size, and the follow-up period. If the articles were ambiguous or lacking of outcomes, we contacted the author. If the author was not available, we extracted the relevant data through consensus.

### Statistical analysis

Data analyses were performed using Version 5.3 Review Manager statistical software. Heterogeneity among studies was assessed using χ^2^ and *I*^2^ statistics. *I*^2^ **≤** 50% suggests that heterogeneity is not statistically significant. *I*^2^ > 50% indicates the presence of heterogeneity, and heterogeneity analysis will be further performed. If there was no significant heterogeneity between studies, the fixed-effect model was used. Otherwise, the random effects model was applied. Publication bias was indicated by a funnel plot. The value *P* < 0.05 was considered to be statistically significant.

## Results

### Search results

**Fig. 1:**
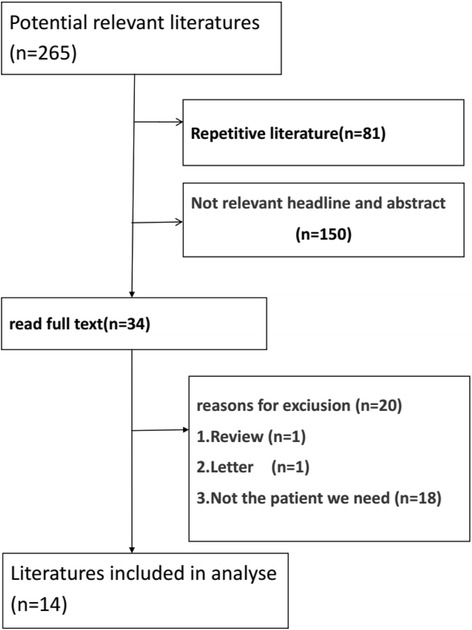
Flow chart of literatures screening and the reasons for exclusion

A total of 265 potential relevant studies were selected from electronic databases, 251 were excluded as they did not meet the inclusion criteria. 14 studies were finally considered for this study. The studies selection process is shown in Fig. 1.

### Study characteristics

Table 1 demonstrates the basic features of the studies. A total of 14 studies from 6 countries were included in our analysis, of which 12 [7–18] were prospective cohort studies, and 2 [19, 20] were retrospective cohort studies. Han [9], He [10] and Ergelen [19] have less than six stars. Akpek [7] and Kaya [13] are multi-country study and the others are single-country study. A total of 10,245 patients were enrolled, including 7908 males. The follow-up time was 3.8 days to 3395 days.

**Table 1:** Characteristics of included studies and quality evaluation of documents

Study	Study	Country	male,%	Age mean(SD)	NLR grouping	Follow-up (days)	Observation index	Quality score
Akpek, [7] 2012	Prospective cohort study	Turkey, China, USA	327(78.0)	59.4 ± =12.4	≤3.3 > 3.3	6.7(mean)	In-hospital: in-stent thrombosis, MACE, nonfatal MI, no reflow, all-cause mortality	6
Arbel, [8] 2014	Prospective cohort study	Israel	436(81.0)	61 ± 13	< 6.5 ≥6.5	1044 (median)	In-hospital: arrhythmia Long-term: all-cause mortality	6
Han, [9] 2013	Prospective cohort study	Korea	247(75.8)	61.9 ± 12.3	≤3.30 3.31–6.52 > 6.53	360(total)	In-hospital: MACE, nonfatal MI, no reflow, all-cause mortality Long-term: nonfatal MI, MACE, all-cause mortality	5
He, [10] 2014	Prospective cohort study	China	546(78.9)	60.27	< 3.16 3.16–4.75 > 4.75	3395 (median)	In-hospital: arrhythmia, no reflow, cardiac mortality, all-cause mortality Long-term: cardiac mortality, all-cause mortality, MACE	5
Her, [11] 2017	Prospective cohort study	Korea	140(81.4)	57.1 ± 12.4	< 5.8 ≥5.8	1230(median)	In-hospital: MACE 12 months follow-up: MACE, Long-term: all-cause mortality, nonfatal MI	6
Park JinJoo, 2013 [12]	Prospective cohort study	Korea	235(72.0)	60.9 ± 13.9	< 5.44 ≥5.44	1092(median)	Long-term: all-cause mortality	6
Kaya, [13] 2013	Prospective cohort study	Turkey, China, USA	535(78.4)	60.8	< 2.3 2.3–4.4 > 4.4	1299(median)	In-hospital: in-stent thrombosis, MACE, non-fatal MI, no reflow, cardiac mortality, Long-term: Non-fatal MI,MACE, cardiac mortality	6
Pan, [14] 2015	Prospective cohort study	China	496(78.0)	59.27 ± 11.27	< 3.0 3.0–6.40 > 6.40	360(total)	In-hospital: angina, arrhythmia, cardiac mortality, 12 months follow-up: all-cause mortality	6
Sen, [15] 2013	Prospective cohort study	Turkey	176(86.3)	55.8	< 3.30 3.30–4.56 > 4.56	1140 (total)	In-hospital: all-cause mortality, MACE, no reflowAt three-year follow up, all-cause mortality, MACE	6
Shen, [16] 2010	Prospective cohort study	China	329(59.7)	61	1.44–3.45 3.45–4.81 4.82–6.46 6.47–22.57	1898 (median)	In-hospital: all-cause mortality Long-term: all-cause mortality	6
Tanriverdi, [17] 2017	Prospective cohort study	Turkey	285(77.4)	59.6	< 5.47 ≥ 5.47	3.8(mean)	In-hospital: all-cause mortality	6
Zuin, [18] 2017	Prospective cohort study	Italy	1724(71.8)	64.5	< 2.1 3.4–4.1 > 4.1	363(median)	Long-term: cardiovascular mortality	6
Ergelen, [19] 2014	Retrospective cohort study	Turkey	2015(83.6)	56.4	≤6.97 > 6.97	630 (median)	In hospital: AHF, MACE, nonfatal MI, no reflow, cardiovascular mortality, Long-term: MACE, nonfatal MI, cardiovascular mortality	5
Gazi, [20] 2015	Retrospective cohort study	Turkey	417(79.9)	62.57	≤5.77 > 5.77	5.7(mean)	In-hospital: AHF, angina, arrhythmia, all-cause mortality, nonfatal MI	6

### Incidence of mortality and cardiovascular events

**Fig. 2:**
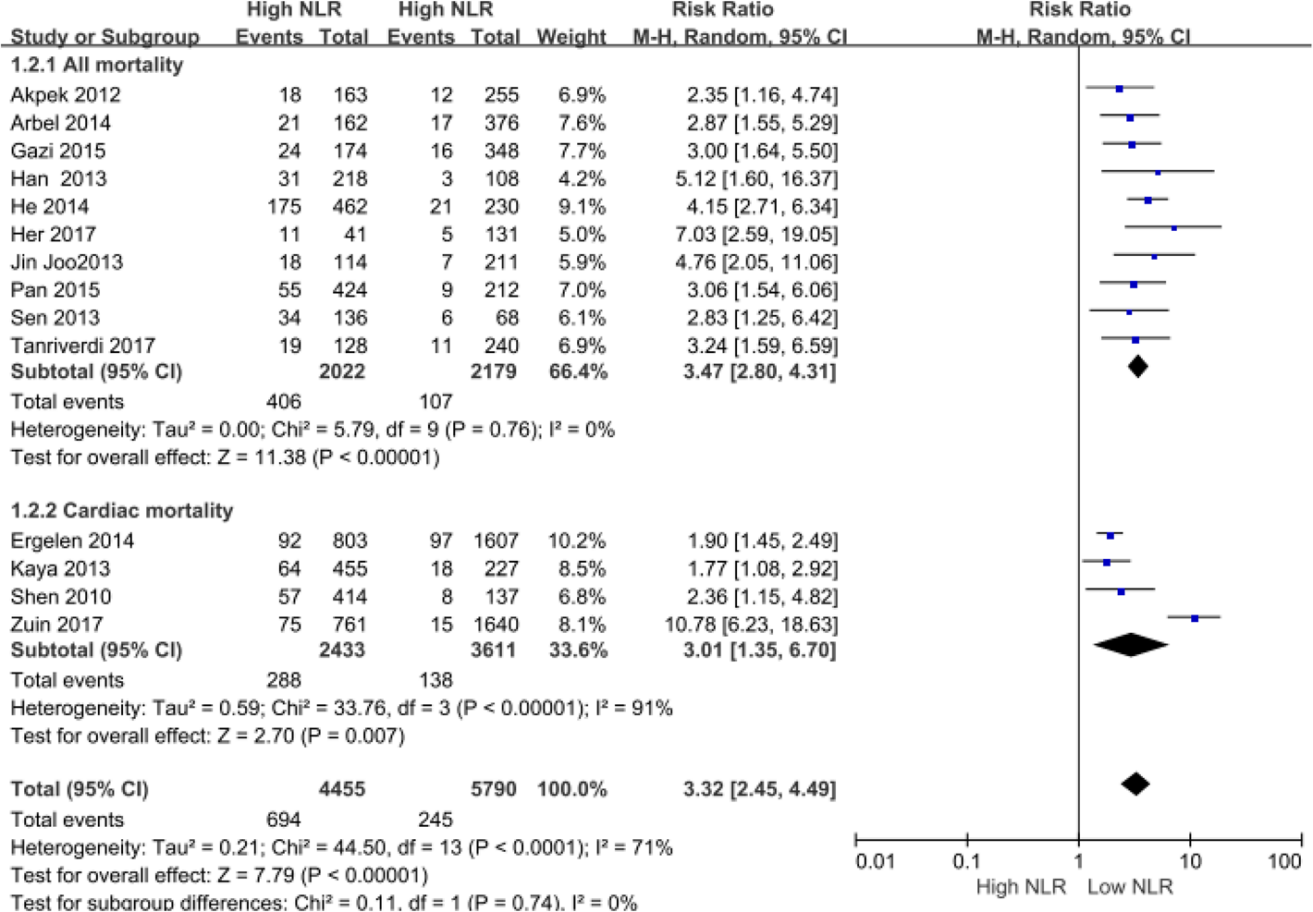
Comparing the mortality between high NLR groups and the low NLR groups

**Fig. 3:**
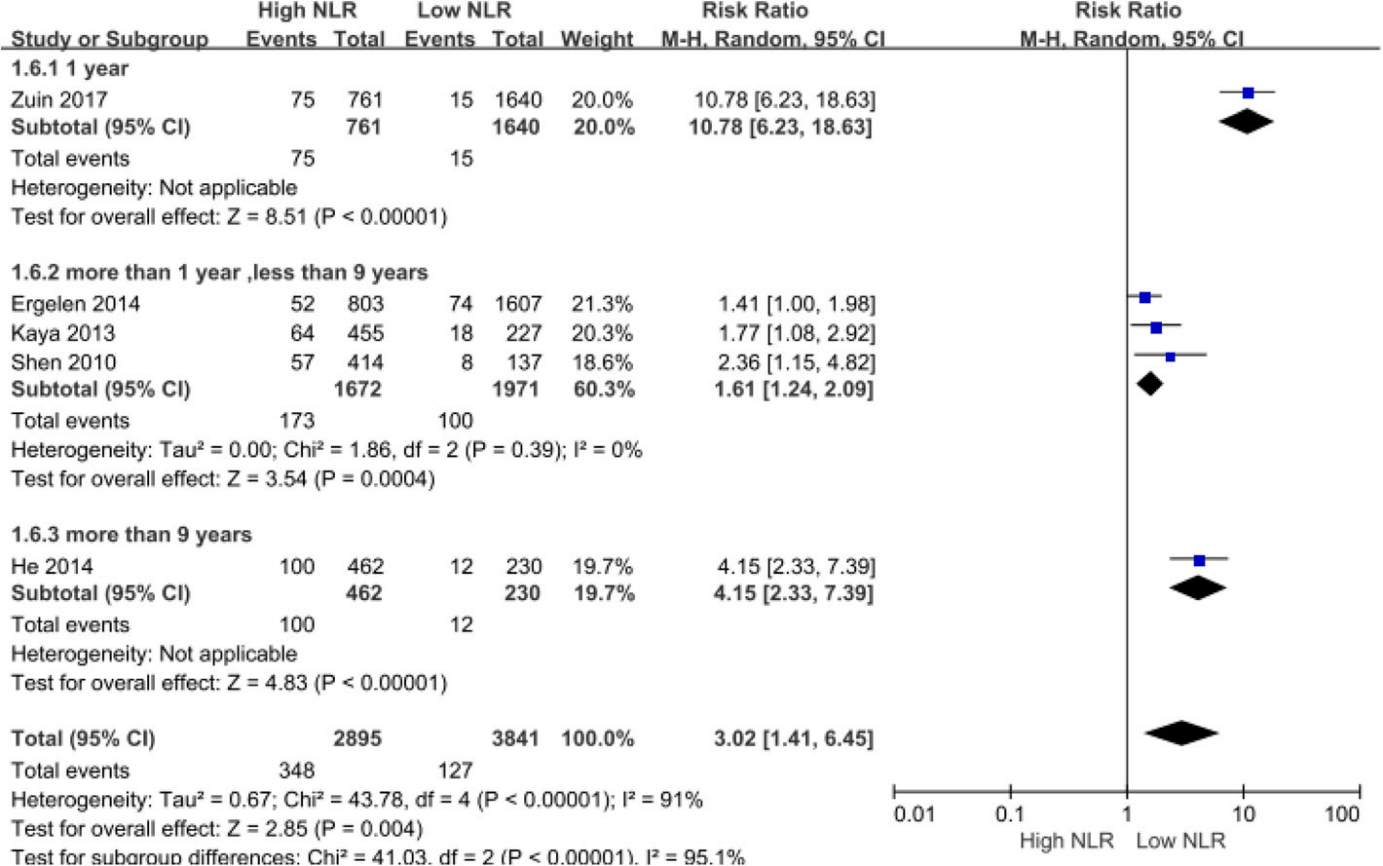
Comparing the long-term cardiac mortality between high NLR groups and the low NLR group

Based on heterogeneity, the fixed effects model was applied to in-hospital cardiac mortality (*P* < 0.001; relative risk (RR) 3.22; 95% confidence interval (CI) 2.25–4.60), in-hospital all mortality (*P* < 0.001; RR3.23; 95% CI 2.28–4.57), long-term all mortality (*P* < 0.001; RR 3.82; 95% CI 2.94–4.96), in-hospital major adverse cardiovascular events (MACE) (*P* < 0.001; RR 2.00; 95%CI 1.62–2.46), in-stent thrombosis (*P* < 0.001; RR2.72; 95% CI 1.66–4.44), in-hospital nonfatal MI (*P* < 0.001; RR 1.93; 95% CI 1.43–2.61), angina (*P* = 0.007; RR 1.67; 95% CI 1.15–2.41), AHF (*P* < 0.001; RR 1.81; 95% CI 1.48–2.21), and arrhythmia (*P* = 0.002; RR 1.38; 95% CI 1.13–1.69). Meanwhile, the random effects model was used for mortality (*P* < 0.001; RR 3.32; 95%CI 2.45–4.49), long-term cardiac mortality (*P* = 0.004; RR 3.02; 95% CI 1.41–6.45), long-term MACE (*P* < 0.001; RR 2.49; 95% CI 1.47–4.23), long-term nonfatal MI (*P* = 0.46; RR 1.32; 95% CI 0.63–2.75), and no reflow (*P* < 0.001; RR 2.28; 95% CI 1.46–3.57). No significant difference was observed for long-term nonfatal MI (*P* = 0.46; RR 1.32; 95% CI 0.63–2.75) (Figs. 2, 3; Table 2, and Additional file 1, Additional file 2, Additional file 3, Additional file 4, Additional file 5, Additional file 6, Additional file 7, Additional file 8, Additional file 9, Additional file 10, Additional file 11, Additional file 12).

**Table 2:** Results of high NLR and low NLR mortality rates and cardiovascular events

Outcomes	Included studies	Heterogeneity			Test for overall effect		
		χ^2^	P	I^2^(%)	Z	P	RR 95%CI
In-hospital							
AHF	19,20	1.54	0.22	35	5.81	< 0.00001	1.81[1.48,2.21]
Angina	**14,20**	0.08	0.78	0	2.71	0.007	1.67[1.15,2.41]
Arrhythmia	8, 10,14,20	0.82	0.84	0	3.14	0.002	1.38[1.13,1.69]
In-stent thrombosis	7,13	0.03	0.86	0	3.99	< 0.0001	2.72[1.66,4.44]
MACE	7,9,11,13,15,19	3.83	0.57	0	6.53	< 0.00001	2.00[1.62,2.46]
Nonfatal MI	7,9,13,19,20	4.88	0.30	18	4.32	< 0.0001	1.93[1.43,2.61]
No reflow	7,9,10,13,15,19	40.00	< 0.00001	87	3.63	0.0003	2.28[1.46,3.57]
All mortality	7,9,10,15,17,20	1.87	0.87	0	6.61	< 0.00001	3.23[2.28,4.57]
Cardiac mortality	10,13,14,16,19	1.77	0.78	0	6.42	< 0.00001	3.22[2.25,4.60]
Long-term							
Nonfatal MI	9, 11,13,19,	10.90	0.01	72	0.75	0.46	1.32[0.63,2.75]
MACE	9,10,11,13,15,19	53.33	< 0.00001	91	3.39	0.0007	2.49[1.47,4.23]
All mortality	8,9,10,11,12,14,15	3.85	0.70	0	10.06	< 0.00001	3.82[2.94,4.96]
Cardiac mortality	10,13,16,18,19	43.78	< 0.00001	91	2.85	0.004	3.02[1.41,6.45]

### Heterogeneity analysis

Analyses with *I*^2^ > 50%, including death, no reflow, long term nonfatal MI, MACE, and cardiac mortality, we conducted a subgroup analysis. The results showed that heterogeneity may come from the time of patient follow- up, race, type of study, etc.

### Publication bias

**Fig. 4:**
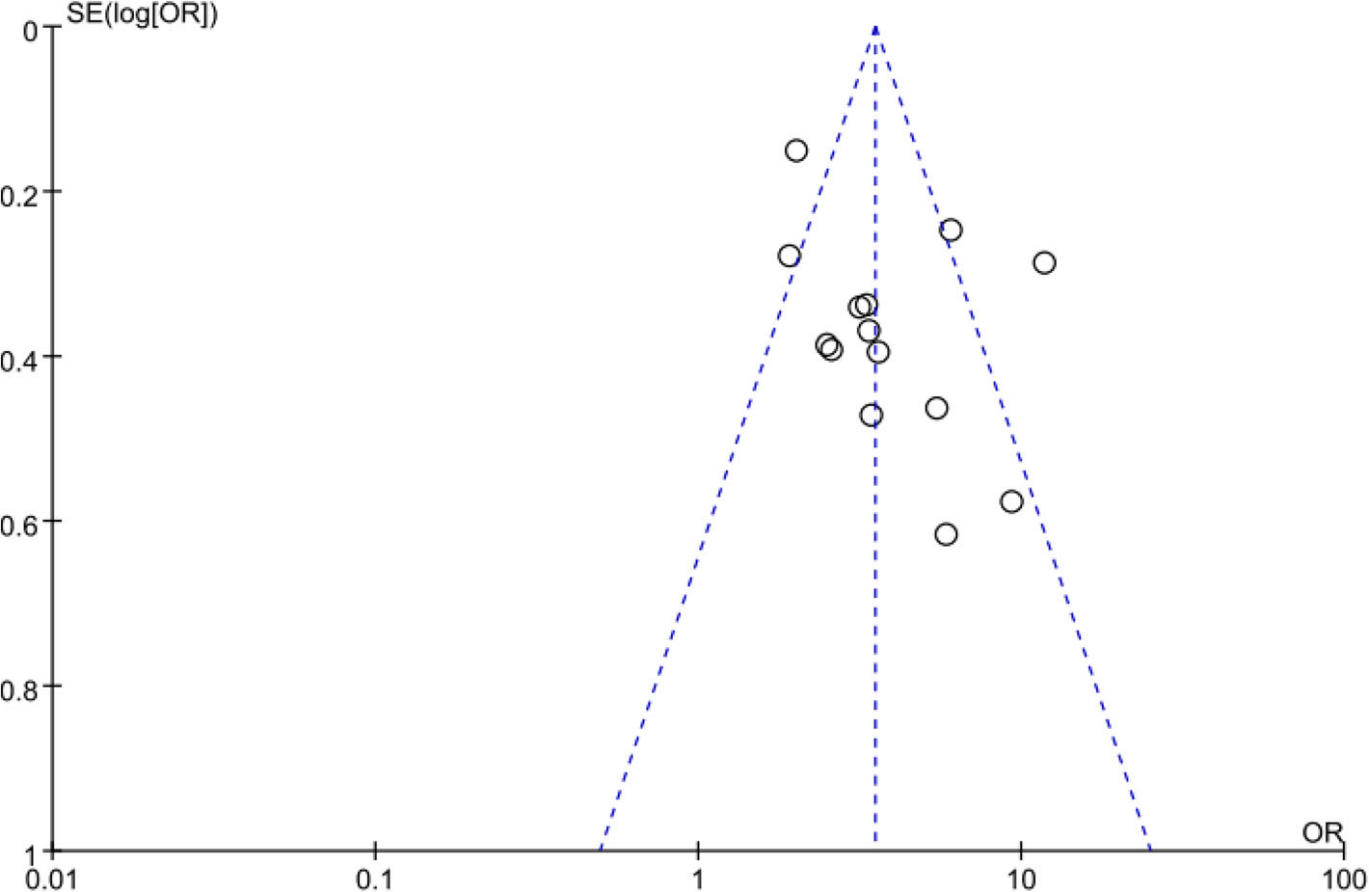
Funnel plot results of 14 studies according to the risk of mortality

No bias was found in the results of 14 studies on funnel plots of mortality risk.(Fig. 4).

## Discussion

The results of our meta-analysis show that the high NLR group has a higher risk of angina, advanced HF, arrhythmia, MACE, cardiac mortality, all mortality, in-stent thrombosis, nonfatal MI, and no reflow than the low NLR group during hospitalization. In addition, the risk of long-term mortality, cardiac mortality and MACE in the high NLR group was higher than that of the low NLR group. However, no significant difference was observed for long-term nonfatal MI.

Wang [21] and Zhang [22] reported a meta-analysis, which discussed the predictive value of NLR in patients after angiography or cardiac revascularization. Similar conclusions were drawn from long-term all-cause mortality and several other results. However, this study is significantly different from their studies with regards to the literature inclusion criteria. The trials they selected did not include all patients with acute STEMI who underwent emergency interventional therapy. The time and method of opening blood vessels may affect the prognosis of patients, so we reduce the impact of these factors.

A large number of experiments have demonstrated that inflammatory response plays an important role in the development and progression of acute coronary syndrome (ACS) [23, 24]. White blood cell plays a vital role in the progression of atherosclerosis and destabilization, leading to thrombotic events [25, 26]. Increased white blood cell count is associated with an increased mortality for STEMI patients [27]. However, the total number of leukocytes is easily influenced by race and sex, and it is difficult to establish a reference range for the whole population [28].

In recent years, in-depth study of the clinical effect of inflammatory reaction in occurrence and development of coronary heart disease observed that different subtypes of leukocytes compared with the total leukocyte count and specific subtype, including neutrophils, lymphocytes and monocytes, have more predictive value in the risk assessment of cardiovascular disease [29].

Neutrophils are the first white blood cell subsets to be observed in the damaged myocardium and move away from the myocardium after phagocytizing debris [16]. Neutrophils play an important role in the destabilization of atherosclerotic plaques [30]. CD4 + T lymphocytes belong to the regulatory arm of the immune system, playing a key role in regulating the inflammatory response at different stages of atherogenesis and acute MI [31–33]. However, neutrophils count is easily affected by the patient’s own state, such as blood volume. The CD4 + T lymphocyte counts are not readily available for immediate blood testing.

In recent years, studies have observed that NLR, by combining the change in neutrophils and lymphocytes in the course of inflammation, is a more powerful predictor of cardiovascular disease than any other leukocyte subtypes [18, 34, 35]. NLR can be obtained from routine blood tests and used as a cost effective biomarker for inflammation. Previous studies observed that NLR as a new inflammatory marker is a good prognostic factor for patients with ACS [36, 37]. Many studies have observed that high NLR levels have a very important relationship with cardiovascular and all-cause mortality in STEMI patients, during hospitalization or long-term [16, 20, 38]. In addition, several studies have shown evidence that a higher NLR is associated with a lower ejection fraction after STEMI [8, 11]. Several studies have also observed that high NLR levels are often associated with complex coronary arteries in patients [39, 40]. Left ventricular apical thrombus and remodeling were also found to be related to high NLR [41, 42].

Hence, NLR as a new marker of inflammation might be for the assessment of risk levels in STEMI patients with PCI. However, in order to reduce the incidence of complications and deaths, active treatment may be needed to reduce NLR levels in patients with STEMI, which requires a more rigorous multi-center clinical trial to confirm. There are several limitations in our analysis. First, less relevant experiments was included. Second, different methods may be used for different cell counts. There is no uniform counting standard. Third, the observation indexes of the experiments are different. Several experiments only provided hospital events. Several studies were followed for a short time, while other experiments used telephone follow-up may affect accuracy of the data. Finally, our analysis failed to correct for interference from a number of factors.

## Conclusions

Our meta-analysis showed that NLR levels were related to the hospital and long-term prognosis of patients with STEMI after PCI.

## Additional files

Additional file 1 The result of advanced HF (*P* < 0.001; RR 1.81; 95%CI 1.48–2.21). (DOCX 41 kb)

Additional file 2 The result of Angina(*P* = 0.007; RR 1.67; 95%CI 1.15–2.41). (DOCX 41 kb)

Additional file 3 The result of arrhythmia (*P* = 0.002; RR 1.38; 95% CI 1.13–1.69). (DOCX 47 kb)

Additional file 4 The result of in-stent thrombosis (*P* < 0.001; RR 2.72; 95%CI 1.66–4.44). (DOCX 41 kb)

Additional file 5 The result of in-hospital MACE (*P* < 0.001; RR 2.00; 95%CI 1.62–2.46). (DOCX 55 kb)

Additional file 6 The result of in-hospital nonfatal MI(*P* < 0.001; RR 1.93; 95%CI 1.43–2.61). (DOCX 51 kb)

Additional file 7 The result of no reflow (*P* < 0.001; RR 2.28; 95%CI 1.46–3.57). (DOCX 95 kb)

Additional file 8 The result of in-hospital all mortality (*P* < 0.001; RR 3.23; 95% CI 2.28–4.57). (DOCX 54 kb)

Additional file 9 The result of in-hospital cardiac mortality (*P* < 0.001; RR 3.22;95% CI 2.25–4.60). (DOCX 51 kb)

Additional file 10 The result of long-term nonfatal MI (*P* = 0.46; RR 1.32; 95%CI 0.63–2.75). (DOCX 77 kb)

Additional file 11 The result of long-term MACE (*P* < 0.001; RR 2.49; 95%CI 1.47–4.23). (DOCX 86 kb)

Additional file 12 The result of long-term all mortality (*P* < 0.001; RR 3.82; 95% CI 2.94–4.96). (DOCX 57 kb)

## Abbreviations

ACS: Acute coronary syndrome
AHF: Advanced heart failure
CI: Confidence interval
MACE: Major adverse cardiovascular events
MI: Myocardial infarction
NLR: Neutrophil to lymphocyte ratio
PCI: Percutaneous coronary intervention
RR: Relative risk
STEMI: ST segment elevation myocardial infarction
