# Supplementary figures and images for: Predictive value of neutrophil to lymphocyte ratio in patients with acute ST segment elevation myocardial infarction after percutaneous coronary intervention: a meta-analysis
Source: BMC Cardiovasc Disord. 2018 May 2;18:75. doi: 10.1186/s12872-018-0812-6 (PMC5930503; doi:10.1186/s12872-018-0812-6)

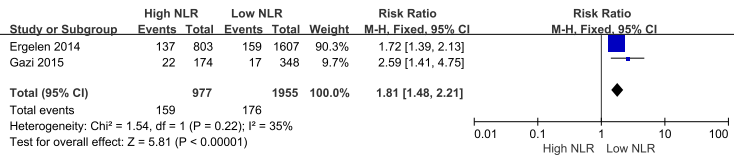

Supplement: Supplementary file 1 — The result of advanced HF (P < 0.001; RR 1.81; 95%CI 1.48–2.21). (DOCX 41 kb) [file 12872_2018_812_MOESM1_ESM.docx]

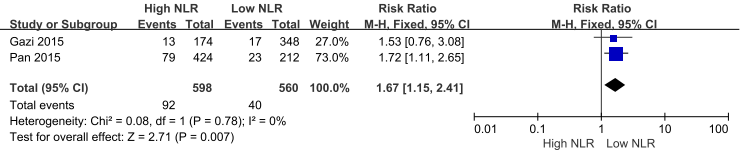

Supplement: Supplementary file 2 — The result of Angina(P = 0.007; RR 1.67; 95%CI 1.15–2.41). (DOCX 41 kb) [file 12872_2018_812_MOESM2_ESM.docx]

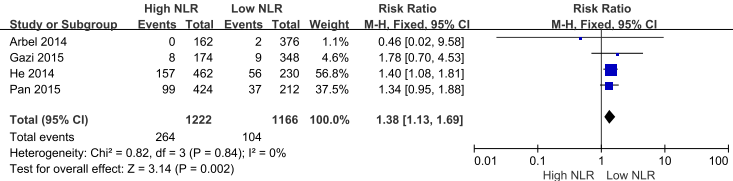

Supplement: Supplementary file 3 — The result of arrhythmia (P = 0.002; RR 1.38; 95% CI 1.13–1.69). (DOCX 47 kb) [file 12872_2018_812_MOESM3_ESM.docx]

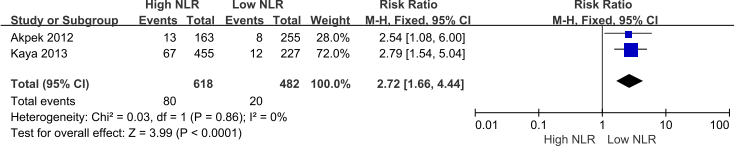

Supplement: Supplementary file 4 — The result of in-stent thrombosis (P < 0.001; RR 2.72; 95%CI 1.66–4.44). (DOCX 41 kb) [file 12872_2018_812_MOESM4_ESM.docx]

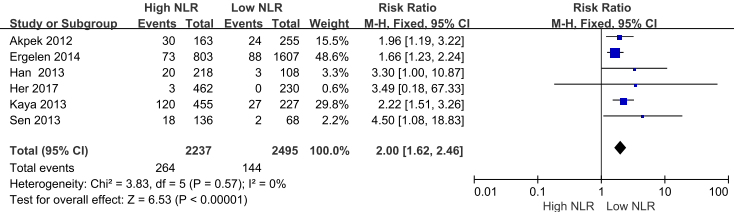

Supplement: Supplementary file 5 — The result of in-hospital MACE (P < 0.001; RR 2.00; 95%CI 1.62–2.46). (DOCX 55 kb) [file 12872_2018_812_MOESM5_ESM.docx]

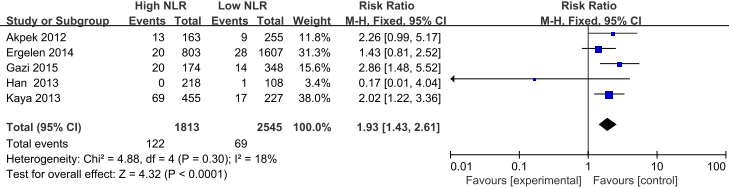

Supplement: Supplementary file 6 — The result of in-hospital nonfatal MI(P < 0.001; RR 1.93; 95%CI 1.43–2.61). (DOCX 51 kb) [file 12872_2018_812_MOESM6_ESM.docx]

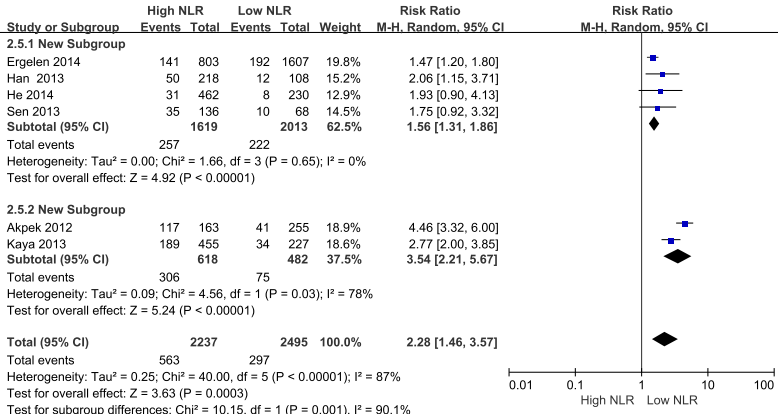

Supplement: Supplementary file 7 — The result of no reflow (P < 0.001; RR 2.28; 95%CI 1.46–3.57). (DOCX 95 kb) [file 12872_2018_812_MOESM7_ESM.docx]

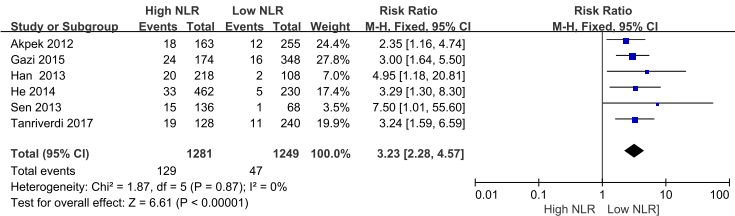

Supplement: Supplementary file 8 — The result of in-hospital all mortality (P < 0.001; RR 3.23; 95% CI 2.28–4.57). (DOCX 54 kb) [file 12872_2018_812_MOESM8_ESM.docx]

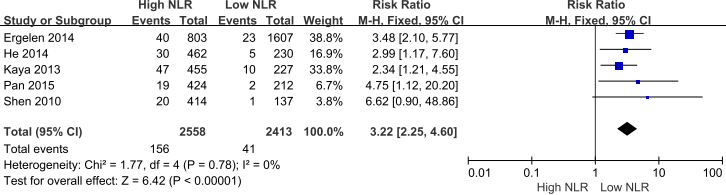

Supplement: Supplementary file 9 — The result of in-hospital cardiac mortality (P < 0.001; RR 3.22;95% CI 2.25–4.60). (DOCX 51 kb) [file 12872_2018_812_MOESM9_ESM.docx]

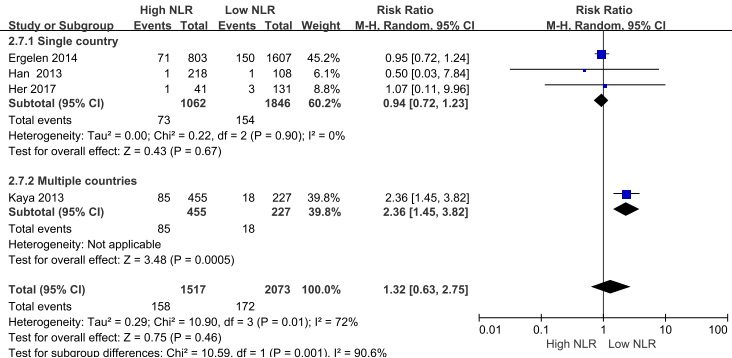

Supplement: Supplementary file 10 — The result of long-term nonfatal MI (P = 0.46; RR 1.32; 95%CI 0.63–2.75). (DOCX 77 kb) [file 12872_2018_812_MOESM10_ESM.docx]

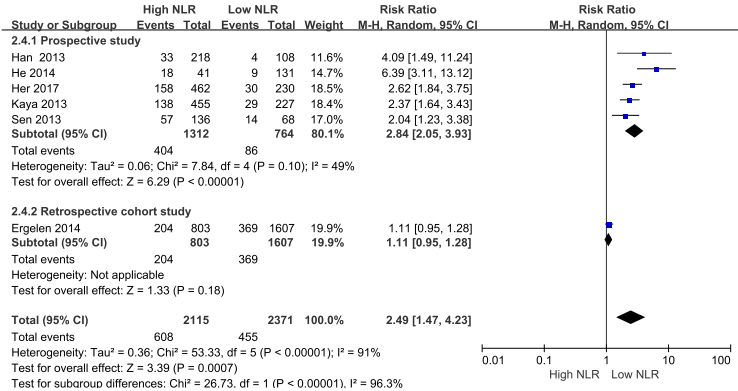

Supplement: Supplementary file 11 — The result of long-term MACE (P < 0.001; RR 2.49; 95%CI 1.47–4.23). (DOCX 86 kb) [file 12872_2018_812_MOESM11_ESM.docx]

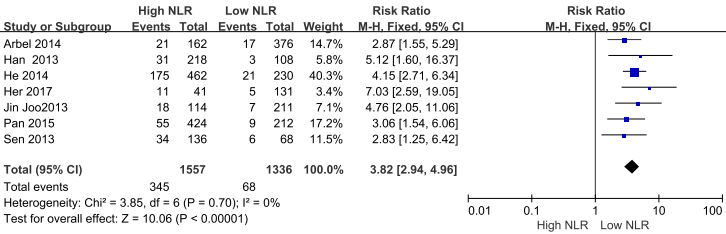

Supplement: Supplementary file 12 — The result of long-term all mortality (P < 0.001; RR 3.82; 95% CI 2.94–4.96). (DOCX 57 kb) [file 12872_2018_812_MOESM12_ESM.docx]
